# Measuring the performance of computer vision artificial intelligence to interpret images of HIV self-testing results

**DOI:** 10.3389/fpubh.2024.1334881

**Published:** 2024-02-07

**Authors:** Stephanie D. Roche, Obinna I. Ekwunife, Rouella Mendonca, Benn Kwach, Victor Omollo, Shengruo Zhang, Patricia Ongwen, David Hattery, Sam Smedinghoff, Sarah Morris, Daniel Were, Dino Rech, Elizabeth A. Bukusi, Katrina F. Ortblad

**Affiliations:** ^1^Public Health Sciences Division, Fred Hutchinson Cancer Center, Seattle, WA, United States; ^2^Audere, Seattle, WA, United States; ^3^Centre for Microbiology Research, Kenya Medical Research Institute, Kisumu, Kenya; ^4^Department of Epidemiology, University of Washington, Seattle, WA, United States; ^5^Jhpiego, Nairobi, Kenya; ^6^Department of Global Health, University of Washington, Seattle, WA, United States; ^7^Department of Obstetrics and Gynecology, University of Washington, Seattle, WA, United States

**Keywords:** artificial intelligence, HIV self-testing, HIV prevention, mHealth, sub-Saharan Africa, differentiated service delivery, Kenya, pharmacy

## Abstract

**Introduction:**

HIV self-testing (HIVST) is highly sensitive and specific, addresses known barriers to HIV testing (such as stigma), and is recommended by the World Health Organization as a testing option for the delivery of HIV pre-exposure prophylaxis (PrEP). Nevertheless, HIVST remains underutilized as a diagnostic tool in community-based, differentiated HIV service delivery models, possibly due to concerns about result misinterpretation, which could lead to inadvertent onward transmission of HIV, delays in antiretroviral therapy (ART) initiation, and incorrect initiation on PrEP. Ensuring that HIVST results are accurately interpreted for correct clinical decisions will be critical to maximizing HIVST's potential. Early evidence from a few small pilot studies suggests that artificial intelligence (AI) computer vision and machine learning could potentially assist with this task. As part of a broader study that task-shifted HIV testing to a new setting and cadre of healthcare provider (pharmaceutical technologists at private pharmacies) in Kenya, we sought to understand how well AI technology performed at interpreting HIVST results.

**Methods:**

At 20 private pharmacies in Kisumu, Kenya, we offered free blood-based HIVST to clients ≥18 years purchasing products indicative of sexual activity (e.g., condoms). Trained pharmacy providers assisted clients with HIVST (as needed), photographed the completed HIVST, and uploaded the photo to a web-based platform. In real time, each self-test was interpreted independently by the (1) client and (2) pharmacy provider, with the HIVST images subsequently interpreted by (3) an AI algorithm (trained on lab-captured images of HIVST results) and (4) an expert panel of three HIVST readers. Using the expert panel's determination as the ground truth, we calculated the sensitivity, specificity, positive predictive value (PPV), and negative predictive value (NPV) for HIVST result interpretation for the AI algorithm as well as for pharmacy clients and providers, for comparison.

**Results:**

From March to June 2022, we screened 1,691 pharmacy clients and enrolled 1,500 in the study. All clients completed HIVST. Among 854 clients whose HIVST images were of sufficient quality to be interpretable by the AI algorithm, 63% (540/854) were female, median age was 26 years (interquartile range: 22–31), and 39% (335/855) reported casual sexual partners. The expert panel identified 94.9% (808/854) of HIVST images as HIV-negative, 5.1% (44/854) as HIV-positive, and 0.2% (2/854) as indeterminant. The AI algorithm demonstrated perfect sensitivity (100%), perfect NPV (100%), and 98.8% specificity, and 81.5% PPV (81.5%) due to seven false-positive results. By comparison, pharmacy clients and providers demonstrated lower sensitivity (93.2% and 97.7% respectively) and NPV (99.6% and 99.9% respectively) but perfect specificity (100%) and perfect PPV (100%).

**Conclusions:**

AI computer vision technology shows promise as a tool for providing additional quality assurance of HIV testing, particularly for catching Type II error (false-negative test interpretations) committed by human end-users. We discuss possible use cases for this technology to support differentiated HIV service delivery and identify areas for future research that is needed to assess the potential impacts—both positive and negative—of deploying this technology in real-world HIV service delivery settings.

## Introduction

Despite significant progress toward global HIV targets, the world is not currently on track to achieve the Sustainable Development Goal of ending AIDS as a public health threat by 2030 (1). As of 2023, only five countries have achieved the UNAIDS 2025 targets for testing, treatment, and viral suppression—known as “95–95–95”—which stand globally at 86% of people living with HIV (PLHIV) knowing their status; 89% of PLHIV on antiretroviral therapy (ART); and 93% of those on ART virally suppressed (2). Similarly, with 1.3 million new HIV infections in 2022 (2) and only 4.3 million people ever-initiated on daily oral HIV pre-exposure prophylaxis (PrEP) (3), significant work will be needed to achieve the UNAIDS 2025 prevention targets to reduce new infections to 370,000 and make PrEP available to 10 million people (4).

Closing these gaps will likely require intensified differentiated service delivery (DSD) strategies to mitigate barriers to accessing and delivering HIV services at clinics, such as HIV-associated stigma, distance, understaffing, and long wait times (5–9). DSD is a person-centered approach recommended by the WHO for both HIV treatment and prevention interventions that aims to simplify delivery; reduce burden on clients, providers, and healthcare systems; and make HIV services more accessible and acceptable to the individuals in need (5). In practice, DSD models often move service delivery outside of traditional health facilities, task-shift delivery to new cadres of providers, and/or incorporate new innovations and technologies, such as electronic adherence monitors, SMS reminders, and decision support tools (10, 11).

HIV self-testing is one innovation that has been underutilized in HIV DSD (12, 13). Despite HIV self-tests (HIVSTs) having high sensitivity (93.6%−100%) and specificity (99.1%−100%) (14–17); increasing recent and frequent HIV testing in diverse populations (18); being largely acceptable to clients (19); and featuring in the national policies of 98 countries (20), implementation has lagged considerably, with only 52 countries routinely implementing HIV self-testing (20). Additionally, the WHO only recently (in July 2023) endorsed HIV self-testing as an additional testing strategy that should be offered at health facilities and one that should be used for PrEP initiation, continuation, and re-starts (21). To date, HIV self-testing has primarily been used as a screening tool, rather than a diagnostic tool, in part, due to concerns that the quality of test administration and interpretation would be lower than standard-of-care rapid diagnostic testing performed by trained HIV testing services (HTS) providers. Of particular concern are false-negatives that could lead to delays in ART initiation and/or incorrect initiation on PrEP, the latter of which carries an increased risk of developing HIV drug resistance (22). Scale-up of DSD models that fully leverage HIVST's accuracy, privacy, and convenience will likely be contingent on assuaging concerns about testing quality.

Computer vision, a field of artificial intelligence (AI) that gleans meaningful information from visual data (e.g., digital images), could potentially assist with ensuring HIV self-testing quality. The peer-reviewed literature includes dozens of examples of AI algorithms that, in research studies, have performed as well as trained healthcare providers at diagnosing conditions based on medical imaging data (e.g., X-rays, CT scans) (23); and in real-world healthcare delivery settings, some proprietary AI algorithms are already being used to assist clinicians with diagnosis (24). A handful of studies have found AI computer vision technology to also perform well at interpreting rapid antigen/antibody diagnostic tests (RDTs) in the form of lateral flow devices. Such tests indicate positivity by producing one or more test lines. Using machine learning, an AI algorithm can be trained on a set of images of completed tests (all of the same brand) to recognize line patterns for positive and negative results; thereafter, when fed an image of a completed test of that same brand, the AI algorithm can make a determination about (i.e., interpret) the test result. Published examples of this use case largely focus on rapid tests for COVID-19 (25–30), with a handful of examples from malaria (31), influenza (32), Cryptococcosis fungal infection (33), and HIV (34). The HIV example comes from Turbé et al. (34), who trained an AI algorithm using 11,374 images of two brands of HIV RDTs: ABON HIV 1/2/O Tri-Line HIV RDT [ABON Biopharm (Hangzhou) Co., Ltd.,] and Advanced Quality One Step Anti-HIV (1&2) Tri-line Test (InTec PRODUCTS, INC.). The training set images had been collected as part of routine household surveillance in KwaZulu-Natal, South Africa, with the RDTs conducted and photographed by trained fieldworkers. To assess the algorithm's performance, 40 RDTs of the same brands were activated using human blood samples. Each completed test was interpreted independently by 5 healthcare providers (2 nurses and 3 community health workers) using traditional visual interpretation. After interpreting the test, each healthcare worker photographed it using a Samsung tablet running an mHealth application. The images were then independently interpreted by the AI algorithm and by a panel of expert HIV test readers. The final dataset included 190 images. Using the expert panel's interpretation as the “ground truth,” the AI algorithm was found to have 97.8% sensitivity (due to 2 false-negatives) and 100% specificity (i.e., 0 false-positives) and outperformed the healthcare worker group, which had 95.6% sensitivity (due to 4 false-negatives) and 89.0% specificity (due to 11 false-positives). The authors acknowledge several limitations of their evaluation (e.g., small sample size) but note the potential of AI computer vision technology to reduce the risk of false-positive and false-negative HIV RDT results.

To date, no study has investigated how well an AI algorithm might perform at interpreting images of HIVSTs conducted in real-world (non-laboratory) settings by clients. Compared to images of HIV RDTs performed by trained healthcare providers or activated by trained lab technicians using human blood samples, images of HIVSTs conducted by real-world clients on themselves might vary in ways that could affect AI algorithm performance. For example, inexperienced self-testers might apply smaller amounts of the blood sample to the test strip, making test lines harder to detect (35). We, therefore, sought to evaluate the performance of an AI algorithm at interpreting images of HIVSTs collected during routine service delivery at private, community-based pharmacies in Kenya. Secondarily, we sought to understand how the AI algorithm performed compared to trained pharmacy providers and clients—two groups of individuals who are legally allowed to conduct HIV self-testing in Kenya—to inform discussions of whether and how the Kenya Ministry of Health (MOH) might incorporate this technology into models of differentiated HIV service delivery.

## Materials and methods

This study is part of a larger, observational study (hereafter, “HIVST Performance Study”) measuring the performance of blood-based HIVSTs, compared to standard-of-care RDTs delivered by certified HTS providers, at private community-based pharmacies—a venue to which the Kenya MOH is interested in expanding HIV services as part of its HIV DSD strategy (36). The methods of this larger study have been described elsewhere (37). Below, we summarize the methods that are pertinent to this present study on AI algorithm performance.

### Study design and setting

This study uses cross-sectional data from the HIVST Performance Study, which was observational in design and conducted at 20 private pharmacies in Kisumu County in western Kenya from March to June 2022. Kisumu has a population-level HIV prevalence of ~18%—one of the highest in the country (38). Pharmacies were eligible to serve as study sites if they were privately owned (i.e., not supported with government funding), operating legally (i.e., currently registered with Kenya's drug regulatory authority), had a back room where HIV testing and counseling could occur in private, and had on staff at least one full-time licensed pharmacist and/or pharmaceutical technologist—two cadres of pharmacy professionals that the Kenya MOH has expressed interest in leveraging for HIV service delivery (36)—who was willing to participate in research activities. We partnered with the Kisumu County Ministry of Health to identify and purposively select licensed pharmacies to serve as study sites (39).

### Participants

Eligible pharmacy providers were ≥18 years old, worked at one of the study pharmacies, and were willing to complete the required training to deliver the intervention. All pharmacy providers attended a one-day in-person training on offering and assisting (if desired by clients) free HIV self-testing to clients purchasing products or services related to sexual and reproductive health (SRH), such as condoms and emergency contraception; conducting and interpreting Mylan blood-based HIVSTs (Mylan Pharmaceuticals Private Limited, India, manufactured in South Africa by Atomo); and providing counseling.

Eligible pharmacy clients were ≥18 years old and self-reported being HIV-negative or not knowing their HIV status, not currently taking PrEP or ART, and engaging (in the past 6 months) in at least one behavior associated with risk of HIV acquisition (e.g., condomless sex) included in Kenya's eight-item Rapid Assessment Screening Tool (40) or having a potential exposure to HIV within the past 72 h. To recruit pharmacy clients, participating pharmacy providers asked those purchasing the above-described SRH products if they would be interested in participating in a research study that was offering free HIVST services to eligible individuals. This being an observational study intended to evaluate outcomes of interest in their natural context, pharmacy client participants self-selected into the study (i.e., the study did not use probability sampling to recruit study participants).

### Training of HIVST expert readers

Twenty HIVST expert readers were contracted by Audere from IndiVillage (Bangalore, India), a B-Corp-certified company that is compliant with US Health Insurance Portability and Accountability Act (HIPAA), the International Organization for Standardization (ISO), System and Organization Controls (SOC), and the General Data Protection Regulation (GDPR). IndiVillage offers dataset creation, annotation, and labeling services for natural language processing and computer vision (41). In addition to the training and experience obtained as an expert test reader for IndiVillage, all of the test readers contracted by Audere for this evaluation completed a two-week training during which they received in-depth instruction specifically on Mylan HIVST result interpretation. To pass this training, attendees were presented with 50 images of Mylan HIVST results and needed to correctly interpret at least 48 (96%) of them. All 20 attendees passed the training; their role is described in the next section.

### Study procedures

**Figure 1:**
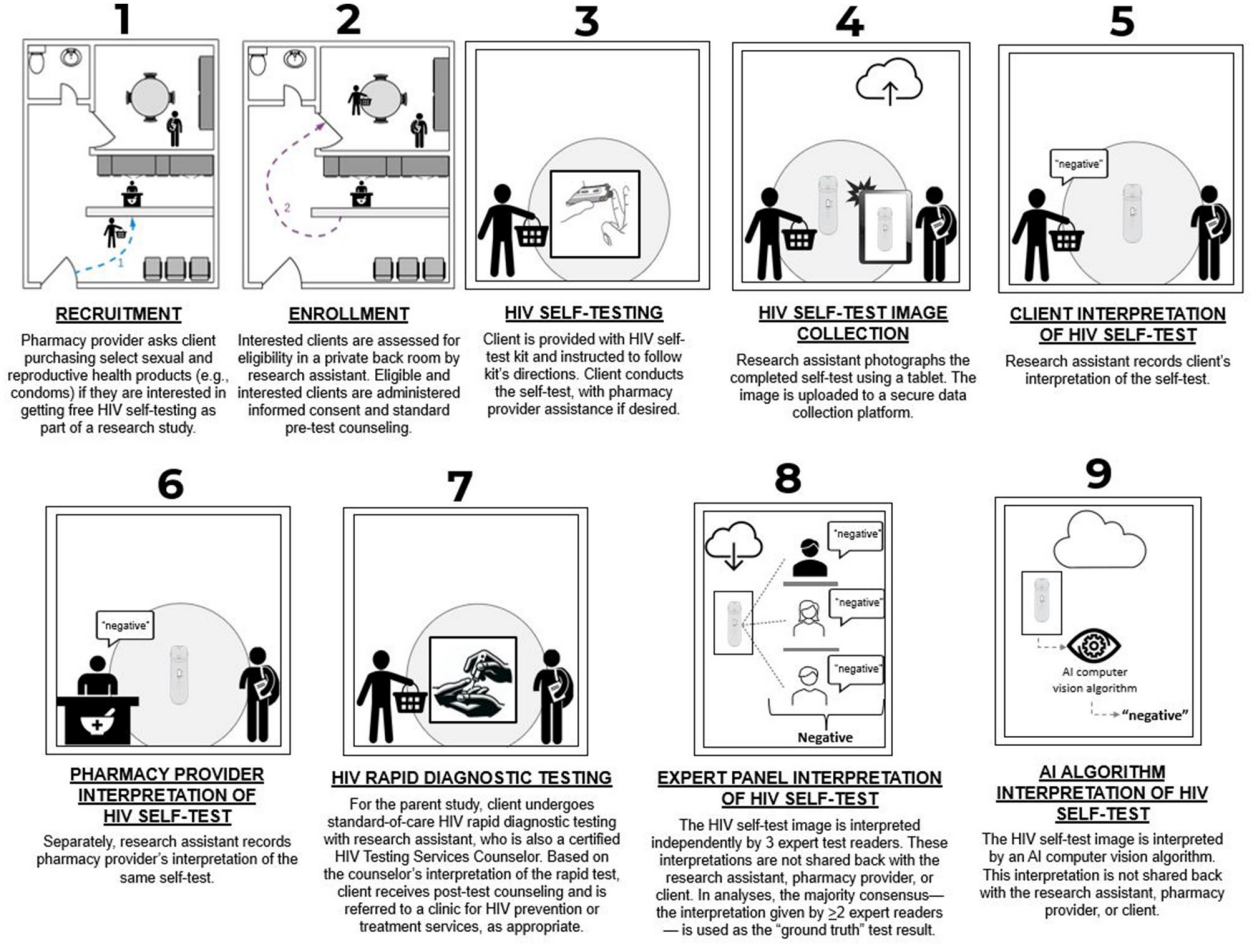
Flow diagram of study procedures.

Figure 1 illustrates the flow of key study procedures in nine steps. As described above, prospective study participants were initially engaged by pharmacy providers (Step 1). In a private back room of the pharmacy, these individuals were assessed for eligibility by a trained research assistant (RA); eligible and interested clients were administered informed consent and provided with pre-test counseling by the RA, who was also a certified HTS provider (Step 2). Pharmacy providers gave clients the option to conduct the HIVST on their own or with their assistance on any aspect of administration except for result interpretation (Step 3). Clients who opted to conduct the HIVST on their own were instructed to follow the directions included in the test kit package and let the pharmacy provider know if they had questions. All HIVSTs were third-generation Mylan blood-based HIVST kits supplied to the pharmacy by the research team.

After the HIVST was completed, the RA photographed it using a Samsung Galaxy A6 tablet, with the image automatically uploaded to the study's secure electronic data collection platform, CommCare (Dimagi, USA) (Step 4). The result was interpreted independently by the pharmacy client (Step 5), then separately by the pharmacy provider (Step 6). These interpretations—which could be “negative,” “positive” or “indeterminant”—were entered into CommCare by the RA. As part of the larger HIVST Performance Study, the client then received standard-of-care HIV rapid diagnostic testing with the RA/HTS provider and, based on the RA/HTS provider's reading of the HIV RDT result, the client received post-testing counseling, was encouraged to consider initiating HIV prevention or treatment services, and was issued a referral slip to a nearby public clinic offering these services for free (Step 7). The RA surveyed each pharmacy provider and client to capture their demographic information.

Within 1 week, each HIVST image was interpreted by a subset of three of the 20 trained HIVST readers (hereafter, “expert panel”) who interpreted each image independently and without access to any additional client data (Step 8). The study treated the expert panel's majority consensus (i.e., the interpretation that two or more of the three HIVST readers gave) as the “ground truth” against which to judge the performance of the AI algorithm (and, secondarily, the performance of the pharmacy client and pharmacy provider). Lastly, each HIVST image was interpreted by an AI algorithm (described in the next section), which also was not provided with any additional client data (Step 9).

### Development of the AI algorithm

A non-profit organization specializing in digital health, Audere (Seattle, USA), developed a platform called HealthPulse AI that leverages AI algorithms for rapid test identification and interpretation. For this study, Audere developed an AI algorithm specifically for interpreting Mylan HIVSTs. Multiple computer vision and machine learning (ML) models comprise the AI algorithm, including (1) an object detector that locates the HIVST and its sub-parts within the image and identifies the HIVST type; (2) a second object detector that examines the HIVST result window (found by the prior object detector) and locates the test and control line regions; (3) a classifier that examines each line region of the result window (found by the second object detector) and outputs line presence probability; and (4) an Image Quality Assurance pipeline that flags adverse image conditions, like blur, low lighting, and overexposure (42). Google's MediaPipe (GMP) framework is used to route images through this sequence of models and return results (43). Customized versions of YOLOX Nano—themselves pretrained on the COCO dataset—are used for both object detectors. A smaller, customized version of MixNet is used as a classifier for the final stage.

To determine how many images the AI algorithm should be trained on to minimize the risk of bias (i.e., the risk of creating an algorithm that only performs well on certain types of images), a comprehensive combinatorics approach was used, taking into consideration possible sources of variation in images when collected in real-world settings, such as environmental conditions (e.g., different lighting, test positioning, image backgrounds); camera quality (affecting, e.g., focal length, image resolution); the test result itself; and test activation (i.e., darkness of lines). Ultimately, it was determined that a reference set of 11,074 images-−6,074 images on which to fit and train the ML models (‘the training set”) and 5,000 images on which to subsequently evaluate the model (“the hold-out set”)—would suffice. The size of this reference set is similar to that used in other studies (34).

The 6,074 images in the training set—which was 82% (*n* = 4,965) negative tests and 18% (*n* = 1,109) positive tests—included 6,048 images of Mylan HIVSTs that had been activated by trained laboratory technicians using human blood samples and 26 images of Mylan HIVSTs featuring faint positive lines that had been conducted by clients on themselves in real-world pharmacy settings. By design, both the training and hold-out sets included images captured under various environmental conditions (as described above) and using the following iOS and Android smartphone devices commonly used in low- and middle-income country settings (44) that vary in camera quality: iPhone 12, SamsungA2Core, Samsung x20, Alcatel U3, Mobicel Geo Trendy Lite, Hurricane Link, Tecno Pop2 mini, Hisense U605, and Ulefone Note 8P. Each image in the reference set was labeled as “positive,” “negative,” or “indeterminant” by 3 human HIVST expert readers, as described above, with the majority consensus used as the “ground truth” result.

After the model was developed, its performance was evaluated using the hold-out set. Based on the intended use case (clinical decision-making support), the a priori accuracy goal for the AI algorithm was a weighted F1 score of at least 95. During the first round of evaluation, the AI algorithm's weighted F1 score was 98.2. Based on this output and performance gaps identified in field deployments, the AI algorithm was further tuned to reduce false-positives and to improve its performance at interpreting images of tests with staining in the result window. The updated model was run against the hold-out set and found to have a weighted F1 score of 98.9. Because the ML models had been trained on 2 mega-pixel images, 2-megapixels—an amount that exceeds the default resolution of most smart devices—was the recommended minimum resolution for optimal performance of the AI algorithm.

### Data analysis

We assessed the performance of the AI algorithm at interpreting the images collected in the HIVST Performance Study on four aspects. First, to understand how well the AI algorithm correctly identified true positives, we calculated sensitivity: the percent of HIVST images classified as “positive” by the expert panel that were interpreted as “positive” by the AI algorithm. Second, to understand how well the AI algorithm correctly identified true negatives, we calculated specificity: the percent of HIVST images classified as “negative” by the expert panel that were interpreted as “negative” by the AI algorithm. Third, to understand how likely it was that the image was truly a negative result if the AI algorithm interpreted it as such, we calculated negative predictive value (NPV): the percent of HIVST images interpreted as “negative” by the AI algorithm that were classified as “negative” by the expert panel. Lastly, to understand how likely it was that the image was truly of a positive result if the AI algorithm interpreted it as such, we calculated positive predictive value (PPV): the percent of HIVST images interpreted as “positive” by the AI algorithm that were classified as “positive” by the expert panel. To assess uncertainty around each of these estimates, we calculated binomial 95% confidence intervals (CI).

To achieve our secondary objective of understanding how well the AI algorithm performed compared to pharmacy providers and clients, we calculated the above-described performance metrics for pharmacy providers and clients. For each pair-wise comparison of interest—the AI algorithm vs. pharmacy providers, and the AI algorithm vs. pharmacy clients—we calculated two performance indices: (1) a sensitivity index, and (2) a specificity index. The former is the ratio of the sensitivity achieved by the AI algorithm to that achieved by the human group of interest; the latter is calculated in the same way except using the values for specificity achieved by the groups of interest. For both performance indices, a value >1 would indicate that the AI algorithm outperformed the human group in question (34). For all analyses, we used Stata v17.0 (StataCorp LLC, USA).

### Ethics

The Kenya Medical Research Institute's Scientific Ethics Review Unit and the Institutional Review Board of the Fred Hutchinson Cancer Center reviewed and approved all study procedures. All pharmacy clients and providers completed written informed consent, which was available in English, Dholuo, and Kiswahili, and were compensated 500 Kenyan Shillings (~$5 US dollars [USD]) for their time completing study activities (e.g., research surveys). The owners of participating pharmacies received 15,000 KES (~$129 USD) per month for use of their space and utilities. Both the expert panel and AI algorithm received only the HIVST image file and were blinded to (i.e., were not provided with) additional contextual information (e.g., client demographics; pharmacy location). To reduce the risk of harm related to HIVST misinterpretation by the AI algorithm, the algorithm's interpretation was not shared back with the client, pharmacy provider, or RA or used for clinical decision-making, with all post-test counseling and referrals to clinic-based HIV prevention or treatment services based on the standard-of-care HIV rapid diagnostic testing conducted by the certified HTS provider.

## Results

### Participants

From March to June 2022, we screened 1,691 pharmacy clients and enrolled 1,500, all of whom completed HIVST. Of 1500 HIVST images uploaded to CommCare, 854 (57%) were of sufficient quality to be interpreted by the AI algorithm (The remaining images had been collected early in the study, prior to adjusting an image resolution setting in CommCare, which compressed the images prior to saving them and reduced their resolution to below the 2-megapixel minimum required resolution to be interpretable by the AI algorithm). Among the pharmacy clients with interpretable HIVST images, 63% (540/854) were female, the median age was 26 years (IQR 22–31), and 39% (335/854) reported having casual sex partners in the past 6 months (Table 1). The majority (75%, 640/854) of clients opted to complete HIVST with some form of assistance from the pharmacy provider. Among 40 pharmacy providers who delivered HIVST and completed a survey, 40% (16/40) were female, the median age was 31 years (IQR 27–31), and 43% (17/40) owned the pharmacy they worked in. The median duration of practice among pharmacy providers was 6 years (IQR 4–10).

**Table 1:** Characteristics of the pharmacy clients and providers.

**Pharmacy client characteristic**	***N* = 854**
Female	540 (63%)
Age, median (IQR)	27 (22, 31)
Average monthly income (±SD), Kenyan Shillings (USD)	15,274 ± 25,109 (131.4 ± 215.9)
**Income source**	
Trade/sales	303 (36%)
Laborer/semi-skilled	221 (26%)
Professional	118 (14%)
Student	108 (13%)
No income	17 (2%)
Other	87 (10%)
**Relationship status**	
Has one primary partner	466 (55%)
Has casual sex partners only	150 (18%)
Has one primary partner and casual partners	185 (22%)
Had more than one new sexual partner in the last 3 months	278 (33%)
Sexual partner is living with HIV	13 (2%)
Sexual partner has other partner(s)	103 (12%)
24.8-1.5,15.5242pt **Pharmacy provider characteristic**	***N*** = **40**
Female	16 (40%)
Age, median (IQR)	31 (27, 37)
Own the pharmacy	17 (42%)
Years in profession, median (IQR)	6 (4, 10)
Days worked at the pharmacy per week, median (IQR)	6 (6, 7)
Have not provided HIV tests prior to study implementation	5 (12%)
Counsel clients before and/or during HIV testing^a^	32 (91%)
Minutes typically spent counseling a client for an HIV test, median (IQR)	20 (15, 43)

IQR, interquartile range; SD, standard deviation; USD, United States dollar. ^a^Denominator out of 35 providers who answered this question.

### Breakdown of HIVST interpretations

**Figure 2:**
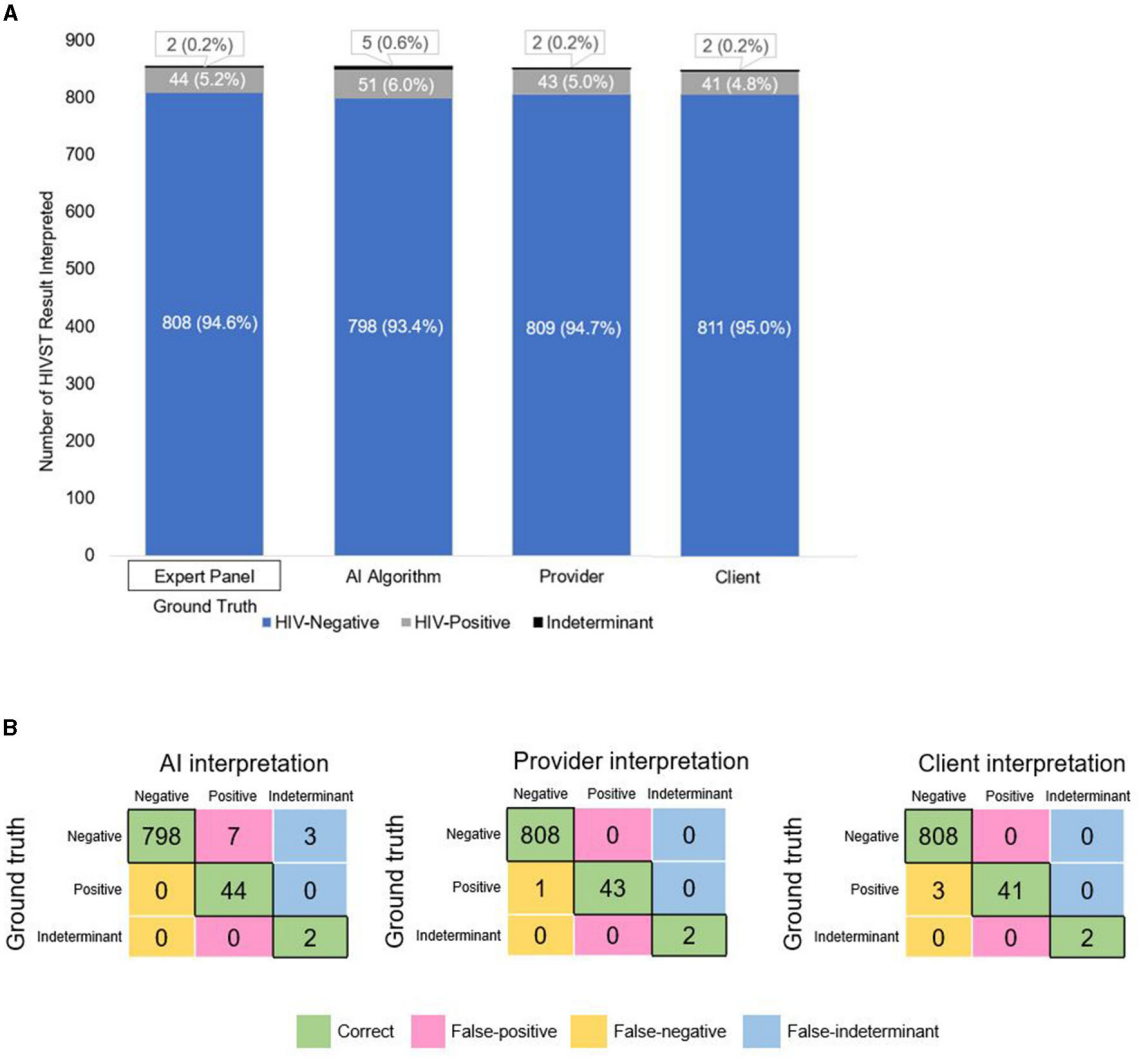
**(A)** Breakdown of HIVST interpretations by expert panel (ground truth), the AI algorithm, pharmacy providers, and pharmacy clients. **(B)** Confusion matrices showing the number of correct, false-positive, false-negative, and false-indeterminant interpretations given by the AI algorithm, pharmacy providers, and pharmacy clients.

Figure 2A shows the proportion of HIVSTs interpreted as negative, positive, and indeterminant by the expert panel, the AI algorithm, pharmacy providers, and pharmacy clients. The expert panel—the “ground truth” for this analysis—classified 95% (808/854) of the images as negative, 5% (44/854) as positive, and 0.2% (2/854) as indeterminant. Overall, this breakdown was similar for the AI algorithm, pharmacy clients, and pharmacy providers. The four performance measures we assessed for each group are listed in Table 2 and described in detail below.

**Table 2:** Performance of an AI algorithm, pharmacy clients, and pharmacy providers at interpreting HIVST results, compared to an expert panel (*n* = 854 HIVST result images).

**Metric^a^**	**AI algorithm interpretation, % (95% CI)**	**Pharmacy client interpretation, % (95% CI)**	**Pharmacy provider interpretation, % (95% CI)**
Sensitivity	100%	93.2% (91.5%, 94.9%)	97.7% (96.7%, 98.7%)
Specificity	98.8% (98.0%, 99.5%)	100%	100%
PPV	81.5% (71.1%, 91.8%)	100%	100%
NPV	100%	99.6% (99.2%, 100%)	99.9% (99.6%, 100%)

AI, artificial intelligence; HIVST, HIV self-testing; CI, confidence interval; PPV, positive predictive value; NPV, negative predictive value. ^a^The reference group for all metrics was the expert panel consensus. Sensitivity and PPV are out of a denominator of 44 HIVST images classified as “positive” by the expert panel. Specificity and NPV are out of a denominator of 808 HIVST images classified as “negative” by the expert panel.

### Specificity and PPV

Correctly identifying HIV-negative individuals as HIV-negative—and minimizing false-positives—saves clients the unnecessary stress and burden of receiving a (false) positive test result and undergoing confirmatory HIV testing. In Figure 2B, the pink- shaded cells of each confusion matrix show the number of false-positive interpretations that each group gave. Focusing on each matrix's top row—which represents the images of HIV-negative tests—we see that the AI algorithm misclassified 10 negative tests-−7 as positive and 3 as indeterminant—and correctly classified 798 of the 808 negative tests as negative, giving it a specificity of 98.8% (95% CI: 98.0%, 99.5%). Because of the 7 false-positive interpretations, the AI algorithm's PPV was 81.5% (95% CI: 71.1%, 91.8%), meaning that if the AI algorithm interpreted a test as positive, there was an ~82% likelihood that the test was truly positive (and, by extension, an ~18% likelihood that the AI algorithm's positive interpretation was incorrect).

By comparison, pharmacy providers and clients correctly classified all 808 negative tests as negative (100% specificity). Neither of these human groups had any false-positives (100% PPV), meaning that if a pharmacy provider or client interpreted a test as positive, there was a 100% likelihood that the test was truly positive.

**Figure 3:**
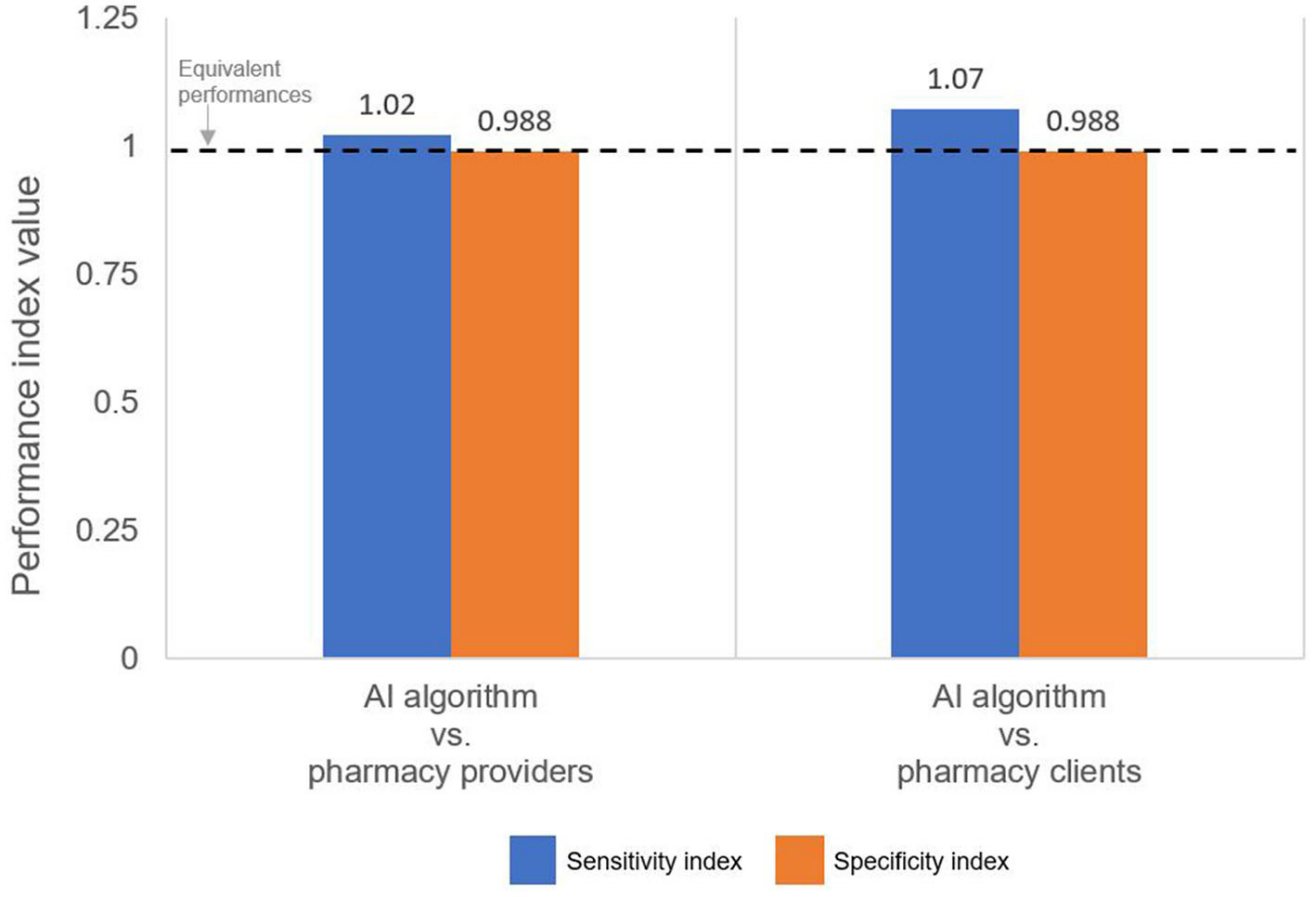
Performance indices comparing the sensitivity and specificity of the AI algorithm to the pharmacy provider and pharmacy client groups. An index >1 indicates that the AI algorithm performed better than the indicated human group.

For both pairwise comparisons of interest—AI algorithm vs. pharmacy provider and AI algorithm vs. pharmacy client—the specificity indices are slightly <1 (Figure 3, orange bar), thus indicating that, compared to both human groups, the AI algorithm was slightly less effective at reading negative tests.

### Sensitivity and NPV

Correctly identifying individuals living with HIV as HIV-positive—and minimizing false-negatives—is arguably even more important, as clients living with HIV who mistakenly believe themselves to be HIV-negative may inadvertently transmit HIV onward to others, experience delayed ART initiation, and/or be inappropriately initiated on PrEP (which has potential implications for developing HIV drug resistance). In Figure 2B, the yellow-shaded cells of each confusion matrix show the number of false-negative interpretations that each group gave. Focusing on each matrix's middle row—which represents the images of HIV-positive tests—we see that the AI algorithm correctly classified all 44 positive tests as positive (100% sensitivity). Because the AI algorithm did not misclassify any positive or indeterminant tests as negative (i.e., no false-negatives), its NPV was 100%, meaning that if the algorithm interpreted a test as negative, there was a 100% likelihood that the test was truly negative.

By comparison, pharmacy providers and clients missed a few positive results, correctly classifying 43 and 41, respectively, of the 44 positive tests as positive; thus, providers and clients had slightly lower sensitivity: 97.3% (95% CI: 96.7%, 98.7%) and 93.2% (95% CI: 91.5%, 94.9%), respectively. Because providers misclassified 1 positive test as negative (i.e., 1 false-negative) and clients misclassified 3 positive tests as negative (i.e., 3 false-negatives), their NPVs were 99.9% (95% CI: 99.6%, 100%) and 99.9% (95% CI 99.2%, 100%), respectively. In other words, if a pharmacy provider or client interpreted a test as negative, there was a 99.9% likelihood that the test was truly negative. Both sensitivity indices are slightly >1 (Figure 3, blue bar), thus indicating that the AI algorithm was slightly more effective at reading positive results than both the pharmacy provider and the pharmacy client groups.

## Discussion

In this study, an AI algorithm trained to interpret images of HIVST results demonstrated perfect sensitivity and negative predictive value (each 100%), 99% specificity, and 86% positive predictive value. Our findings are notable for two main reasons. First, our evaluation found that the AI algorithm did not miss a single HIV infection and, importantly, outperformed humans at correctly identifying positive tests as positive. This finding is significant because false-negatives—i.e., missed diagnoses of HIV infection—are detrimental to the affected individuals and undermine efforts to control the HIV epidemic. Clients who receive false-negative results may unknowingly transmit their HIV infection onward to sex partners, and potentially lose precious time in initiating HIV treatment critical to slowing the progression of the HIV virus (45). Such clients may also be incorrectly initiated on antiretroviral-based HIV prevention drugs (e.g., PrEP)—a scenario that could lead to drug resistance (22) and make the client's HIV infection more difficult and more expensive to treat (46).

Many high HIV burden countries are striving to reach the UNAIDS target of 95% of PLHIV knowing their status (4); however, as countries approach this target, the more difficult and more labor-intensive it becomes to find the remaining, often hard-to-reach individuals (47). Because countries spend millions of dollars each year on HIV case-finding (48), misinterpretation of any such cases is an expensive failure (49, 50). Human error in interpreting positive HIV test results—particularly ones with weak positive lines—is a known and well-documented issue, and many of the proposed interventions to mitigate this issue (e.g., more specialized training for healthcare providers, use of a second test reader) require additional human resources (13, 51). In our study, the AI algorithm detected 4 HIV infections missed by pharmacy providers and pharmacy clients, possibly due to the AI's superior ability to detect faint lines compared to the unassisted human eye. While 4 additional cases of HIV infection detected may appear modest, HIV testing occurs at large scale globally, with over 100 million HIV tests conducted annually worldwide (52). Given this, our study findings give reason for cautious optimism that such AI computer vision technology could potentially help countries improve their HIV case-finding without requiring them to invest significant additional human resources, with the caveat that the exact value such technology provides will necessarily depend on numerous factors, including how commonly used the AI algorithm's specific brand of HIV test is; the existing rate of interpretation error among HIV test users; and the extent to which the test images being fed to the AI algorithm differ from the set on which it was trained (i.e., AI bias, discussed in more detail below).

Our second notable finding is that when the AI algorithm erred, it erred in the more conservative direction, producing some false-positive—but zero false-negative—interpretations. For any diagnostic test, perfect accuracy is virtually impossible to achieve due to factors such as variation in specimen quality; as such, there is a necessary trade-off between sensitivity and specificity (53). Recognizing this, global- and country-level clinical protocols for diagnosing HIV infection (12, 54) first utilize more sensitive HIV tests as first-line assays to weed out most HIV-negative individuals, followed by more specific HIV tests as second- and third-line assays to eliminate false-positives among those who tested positive (55). In our study, the AI algorithm gave seven false-positive interpretations, most likely due to image quality issues (e.g., blurriness), and was outperformed on this metric by the pharmacy client and provider groups, both of which gave zero false-positive interpretations. False-positive interpretations are, indeed, unfavorable, and can lead to adverse consequences for clients, such as anxiety and psychological distress (56); but from a public health perspective, these harms are considered less alarming than those of false-negatives, in part, because of the aforementioned three-test clinical protocols in place that nearly always catch false-positives on second- or third-line assays (57). In short, in the absence of a tool that can interpret HIV tests with perfect sensitivity and perfect specificity, the next-best scenario is, arguably, a tool with perfect sensitivity, deployed with contingency plans for handling its imperfect specificity (e.g., clear warnings to end-users that positive interpretations could be incorrect and should be confirmed via additional testing). For this reason, our study findings give reason for cautious optimism about AI computer vision technology and its potential to support HIV test interpretation. Below we discuss future areas of research; key ethical, financial, and legal considerations for this technology; and study limitations.

### Future areas of research

Whether any HIV program chooses to incorporate AI computer vision technology into its service delivery and, if so, how will depend on a number of context-specific factors, such as how well the algorithm in question performs; how HIV service delivery is structured, staffed, and funded; internet connectivity; local laws regulating the use of AI; and the technology's acceptability to and usability by target end-users. With this caveat in mind, there are a number of potential use cases for AI computer vision technology within HIV service delivery that are worthy of further consideration—and additional research—by HIV stakeholders working in collaboration with AI experts and technology ethicists. These research areas—summarized in Table 3 as Examples A through F and discussed in further detail in the Supplementary material—touch upon potential uses of AI computer vision technology to support the following aspects of HIV service delivery: quality assurance and supported implementation; commodity accountability; and provider training and evaluation. The use cases focus specifically on models of differentiated HIV service delivery (DSD), which—as previously explained—often move service delivery outside of traditional health facilities, task-shift delivery to new cadres of providers, and/or incorporate new technologies or innovations (5).

**Table 3:** Future areas of research related to AI computer vision in HIV differentiated service delivery (DSD).

**Potential uses of AI**		**Relevance for HIV differentiated service delivery (DSD)**
**Area 1: Quality assurance and supported implementation**		
*Help verify test results*	**Example A:** After conducting an HIV test (or self-test), providers (or clients) upload a photo of the result to a digital platform. On the back end, an AI algorithm also interprets the result and notifies parties responsible for overseeing quality of care of any discrepancies, creating an opportunity to change course of action, if needed.	Having a reliable way to verify that HIV tests are correctly interpreted might convince policymakers to allow delivery models that task-shift HIV testing to new cadres of healthcare providers, lay providers, or clients themselves as part of broader DSD efforts to make more efficient use of existing health resources, lower client wait times, reduce visit burden, and/or expand HIV services to new delivery venues.
*Guide and reassure end-users*	**Example B:** Healthcare providers are given the option to compare their interpretation of the HIV test result to that of the AI algorithm.	Because many DSD models engage individuals not previously involved in HIV service delivery, their success hinges on developing end-user knowledge, skills, and self-efficacy to engage in services as intended (e.g., to correctly and confidently conduct HIV tests). AI algorithms could support providers by offering a “second opinion” on result interpretation, giving providers the opportunity to double-check their interpretation of the test prior to making a final determination about the result.
*Assess fidelity*	**Example C:** An AI algorithm is trained to detect possible indications of HIV self-test (HIVST) misadministration—such as the blood sample being placed incorrectly (e.g., in the results window) and test end-users not waiting the recommended duration of time prior to interpreting the result—and flag such cases for further review.	Mechanisms for assuring the quality of HIVST administration and interpretation may make policymakers more willing to support HIV service delivery models that use HIVST in lieu of, or as an additional testing option to, HIV rapid diagnostic testing (RDT). This would benefit clients who prefer HIVST and create potential opportunities to move HIV service delivery, or select parts of it, outside of traditional healthcare settings (See telehealth PrEP model in next row).
*Provide quality control*	**Example D:** An AI algorithm checks the quality of an HIV test result photo uploaded to a digital platform. If a photo does not meet a prespecified quality standard, the user is prompted in real-time to re-take it (while the results are still valid).	Telehealth PrEP delivery models involve remote clinicians using digital data for clinical decision-making. Ensuring that user-submitted digital data is of sufficient quality to be usable could potentially help avoid incorrect clinical decisions and reduce inefficiencies (e.g., time delays) related to data re-collection.
**Area 2: Provider training and evaluation**		
*Evaluate providers*	**Example E:** After an individual completes HTS training, an AI algorithm is run on images from the first 100 HIV tests they conduct, with any discrepancies with the algorithm's interpretation flagged for review.	Compared to using human auditors, assessing HTS provider performance remotely and in an unannounced fashion may be more reliable, cost-saving, have better privacy for clients, and enable regulators to quickly identify providers in need of further training and support.
**Area 3: Commodity accountability**		
*Mitigate fraud*	**Example F**: A country's ministry of health or a donor agency agrees to support HIV service delivery in a private-sector setting by providing commodities (e.g., HIV test kits, PrEP drugs). For accountability purposes, providers are required to write unique client identifiers on used commodities and upload photos of them. An AI algorithm is trained to detect potential signs of fraud, such as upload of duplicate photos and erasure of the unique client identifiers (to re-use test kits).	To date, few countries with high HIV burden have partnered with the private sector to deliver HIV services at scale, in part, because the necessary legislation and systems for cross-sector service delivery (e.g., health information system, supply chain) are not yet in place. Used as a fraud mitigation strategy, AI computer vision could potentially provide an extra measure of accountability that might address policymaker and donor hesitation to support private sector-based HIV DSD models.

AI, artificial intelligence; DSD, differentiated service delivery; HIVST, HIV self-testing; RDT, HIV rapid diagnostic testing; HTS, HIV testing services; PrEP, pre-exposure prophylaxis; RDT, rapid diagnostic testing; HTS, HIV testing services.

### Ethical, financial, and legal considerations

How to ethically implement AI computer vision technology and sustainably finance its development and long-term use at scale while still ensuring that it complies with local laws, are three fast-evolving areas that will determine whether, how, and the pace at which this technology is incorporated into HIV service delivery. Regulations and guidelines around the use of AI for health are still largely nascent (58, 59). Similar to other health services, AI-supported delivery of HIV services will require guardrails not only to ensure that the technology complies with patient privacy and data security laws, but also to handle situations in which the AI returns inaccurate information, especially if inaccuracies are more prevalent for certain groups of individuals due to bias in the data on which the AI was trained. The growing literature on bias in AI machine learning for medicine highlights the importance of and need for bias mitigation strategies and for any deployment of AI to be accompanied by routine evaluations of its performance and impact on patients and providers (60–63). Thinking through the potential ethical issues (59) (e.g., inequitable access to AI technologies exacerbating existing health disparities) and liability risks (e.g., the risk of client self-harm after receiving a false-positive result from an AI algorithm)—and deciding on risk mitigation measures (e.g., limiting client exposure to AI; deciding the content of user agreements for HIVST apps)—will influence this technology's incorporation into HIV service delivery, which will undoubtedly vary by setting and use case.

Similarly, financing AI computer vision algorithms is bound to take many forms. There are three primary costs to consider: (1) the development of the algorithm (a one-time cost); (2) the integration of the algorithm into a digital platform, such as an app or electronic medical record system (also a one-time cost); and (3) the operating costs of running and maintaining the algorithm (an ongoing cost). The development of the algorithm assessed in this study was funded by a private philanthropic organization as a global public good. As such, the governments of low- and middle-income countries can obtain the algorithm from the parent company free of charge. These governments, in turn, are responsible for covering the one-time cost (typically ~$10,000 USD) of integrating the algorithm into their digital health platform of choice. Lastly, governments and/or donors need to budget for the ongoing operating costs, the amount of which would depend on the scale at which the algorithm is used. For example, early data from pilot studies suggests that, when deployed at scale, the cost of running the algorithm could be kept as low as a few cents per test image assessed. Moreover, if an algorithm is deployed in multiple settings (e.g., multiple countries), then the cost for routine algorithm maintenance could be shared, with updates pushed to all end-users. Specific approaches to cost-sharing the development and maintenance of AI computer technology among governments, donors, third-party payers (e.g., private health insurers), and clients is an area for further investigation.

### Study limitations

This study has limitations. First, because this study was conducted within a larger study on HIVST performance, pharmacy providers received comprehensive training on conducting and interpreting HIVST and pharmacy clients were given the option to receive provider assistance conducting HIVST. These factors may have increased the performance of pharmacy clients and providers, thus underestimating the degree to which an AI algorithm might outperform these human groups at HIVST interpretation. Second, due to the previously described error on behalf of the research team early on during data collection whereby an image resolution setting was not adjusted in the electronic data collection platform, the first 646 HIVST images collected during the study did not meet the 2-megapixel minimum required resolution prespecified by the algorithm developer; as such, 646 of the 1,500 total images collected (43%) were excluded from this evaluation. Although our remaining sample size (*n* = 854) was still robust, this may have given the provider group a slight advantage if their performance interpreting the tests whose images were discarded (collected early on in implementation when providers may have still been honing their test interpretation skills) was lower than it was for the tests included in our final analysis. Third, like all observational studies that do not employ probabilistic sampling, our pharmacy provider and client groups—and their respective performances at interpreting HIVSTs—may not be representative of all pharmacy providers and clients; as such, our findings about the performance of those two human groups are not generalizable to other pharmacy providers and clients in Kisumu County or, more broadly, Kenya and similar settings. Lastly, because our study assessed only one AI algorithm trained on a single brand of HIVST kits and only on images of at least 2-megapixel resolution, our findings are not necessarily generalizable to other AI algorithms, types of HIVSTs, or to images below 2-megapixel resolution. However, because the Mylan HIVSTs used in this study have the same general format of many other common biologic tests (e.g., positive results are indicated by two lines), our study findings may be a reasonable indicator of how this technology might perform on other similar tests.

## Conclusions

AI computer vision technology shows promise as a quality assurance tool for HIV testing. Such technology may be especially useful for enabling HIV services to be delivered outside of traditional healthcare settings, by new cadres of providers, and/or at different cadences to better meet client needs and preferences and to use existing health resources more efficiently. Future research could measure the effect size of making AI algorithm result interpretations available to end-users in real-time compared to a control group unassisted by AI (e.g., effect on rate of false-negatives, on provider confidence); assess the feasibility, acceptability, and unintended consequences of using this technology (e.g., ethical issues related to AI interpretation errors, patient privacy, and healthcare worker job security)—among different end-user groups (e.g., community health workers) and in different settings; and conduct cost-effectiveness studies (e.g., quantify the cost per additional case of HIV identified). Future research should also explore AI biases, with an eye toward minimizing biases that may compromise care quality, fairness, and equity. Stakeholders of HIV service delivery should carefully consider leveraging AI computer vision technology as part of broader efforts to make services more client-centered and expedite progress toward HIV epidemic control.

## Data availability statement

The raw data supporting the conclusions of this article will be made available by the authors, without undue reservation.

## Ethics statement

The studies involving humans were approved by the IRB of Fred Hutchinson Cancer Center and the Scientific Ethics Review Unit of the Kenya Medical Research Institute. The studies were conducted in accordance with the local legislation and institutional requirements. The participants provided their written informed consent to participate in this study.

## Author contributions

SR: Formal analysis, Writing—original draft, Writing—review & editing. OE: Formal analysis, Writing—review & editing, Writing—original draft. RM: Software, Project administration, Writing—review & editing. BK: Supervision, Investigation, Project administration, Writing—review & editing. VO: Investigation, Project administration, Supervision, Writing—review & editing. SZ: Data curation, Formal analysis, Writing—review & editing. PO: Resources, Supervision, Project administration, Writing—review & editing. DH: Software, Writing—review & editing. SS: Software, Writing—review & editing. SM: Conceptualization, Software, Project administration, Writing—review & editing. DW: Resources, Supervision, Project administration, Writing—review & editing. DR: Software, Conceptualization, Funding acquisition, Writing—review & editing. EB: Methodology, Supervision, Conceptualization, Funding acquisition, Writing—review & editing. KO: Conceptualization, Funding acquisition, Investigation, Methodology, Supervision, Writing—review & editing.
