# Supplementary material for: Measuring the performance of computer vision artificial intelligence to interpret images of HIV self-testing results
Source: Front Public Health. 2024 Feb 7;12:1334881. doi: 10.3389/fpubh.2024.1334881 (PMC10880864; doi:10.3389/fpubh.2024.1334881)
Supplement: Supplementary file 1 [file Data_Sheet_1.docx]

Supplementary Material

**Table S1. List of acronyms used**

| **Acronym** | **Meaning** |
| --- | --- |
| AI | Artificial intelligence |
| ART | Antiretroviral therapy |
| CI | Confidence interval |
| cRCT | Cluster randomized controlled trial |
| DSD | Differentiated service delivery |
| GDPR | General Data Protection Regulation |
| HIPPA | US Health Insurance Portability and Accountability Act |
| HIVST | HIV self-test |
| HTS | HIV testing services |
| ISO | International Organization for Standardization |
| IQR | Interquartile range |
| ML | Machine learning |
| MOH | Ministry of Health |
| NPV | Negative predictive value |
| PLHIV | People living with HIV |
| PPV | Positive predictive value |
| PrEP | Pre-exposure prophylaxis |
| RA | Research assistant |
| RDT | Rapid diagnostic test |
| SMS | Short message system |
| SOC | System and Organization Controls |
| SRH | Sexual and reproductive health |
| UNAIDS | Joint United Nations Programme on HIV/AIDS |
| USD | United States dollar |
| WHO | World Health Organization |

**Additional description of future research areas for AI computer vision technology for HIV differentiated service delivery**

### Quality assurance and supported implementation

An AI computer vision algorithm similar to the one evaluated in this study is currently being deployed as a quality assurance tool for HIV rapid diagnostic test interpretation in a cluster randomized controlled trial (cRCT)—the Pharm PrEP cRCT—that is delivering PrEP and PEP via 60 private pharmacies in central and western Kenya (**Table 3, Example A**).^1^ In this study, clients in the intervention arms undergo traditional HIV rapid diagnostic testing at the pharmacy with a trained healthcare provider. Clients and providers are shielded from the AI’s interpretation (i.e., the AI is not used by pharmacy providers for clinical decision-making); however, the study implementers receive the AI algorithm’s interpretation and can use it to identify possible cases of misinterpretation and take action accordingly (e.g., review the HIV test image; if necessary, arrange for the client to undergo repeat HIV testing).

Similar AI algorithms have been deployed as clinical decision-making support tools in the ePrEP Kenya pilot—a pilot study of PrEP and PEP delivery via a single online pharmacy in Nairobi County ^2^ —and in the POP INN Clinic ePrEP Program, which is delivering PrEP services to key populations via a partnership between an online pharmacy and 5 health clinics spread across 3 provinces of South Africa.^3, 4^ In both, prospective PrEP or PEP clients complete an HIVST at home and upload an image of the test to an online platform. The test image and the AI’s interpretation are shared with a remote clinician who also interprets the test image and has ultimate say over whether the client is medically eligible for PrEP or PEP (**Table 3,** **Example B**). Clients deemed eligible for PrEP or PEP are issued a prescription, and the medication is courier-delivered to them. Importantly, prior to deploying the AI algorithm, clinicians receive training about the AI algorithm to ensure they understand that the AI’s interpretation is not always correct and about clinical protocols around the AI’s use, which mandate that clinicians make their own determination about the HIV test result and not base clinical decision-making solely on the AI’s interpretation. Future research is needed to assess the effects, both intended and unintended, of this technology when deployed as a quality assurance and/or clinical decision-making support tool.

Also worthy of future research is using AI computer vision technology for fidelity assessments. Both the Pharm PrEP cRCT^5^ and the POP INN ePrEP Program^3, 4^ have deployed AI algorithms to help detect signs of possible HIV test misadministration, such as incorrect application of the blood sample or the end-user not waiting the requisite amount of time for the test to process before interpreting it (**Table 3,** **Example C**). The POP INN ePrEP Program is further using an AI algorithm to check that the digital data being used for remote clinical decision-making—the HIV self-test images—meet prespecified quality standards. If the test image uploaded to the online platform does not meet these standards (e.g., resolution is too low), the AI algorithm flags this, and the client is prompted to re-take the image (**Table 3,** **Example D**). By ensuring that digital data meet quality standards, AI algorithms might increase efficiency by eliminating the task (and associated time delay) of asking clients to re-collect the data. Additional research is needed to evaluate this use case and assess its effects.

### Provider training and evaluation

AI computer vision technology could potentially support training and evaluation of individuals newly trained on HTS or on new types of HIV tests (**Table 3,** **Example E**). For example, when countries update their HIV testing algorithms—like Kenya will do in 2024^6^—such technology could potentially reduce the burden of re-training the entire HTS workforce^7^ by enabling some portion of provider competency evaluation to occur remotely. Using an algorithm to audit early provider performance in this way might also enable regulators to quickly, reliably, and cheaply identify individuals in need of further training and support. Additional research should explore this potential use case.

### Commodity accountability

To expediate progress towards ending the HIV epidemic, some governments are trying to increase HIV service delivery via the private health sector through public-private partnerships; however, a key concern about such partnerships is the lack of cross-sector systems for effectively tracking and accounting for services rendered will lead to fraud.^8, 9^ This is another area where HIV computer vision technology could potentially help and should be researched further. The Pharm PrEP cRCT, for example, which is using PrEP and PEP drugs and HIV RDT kits donated by the Kenya Ministry of Health, requires pharmacy providers to write a unique client identifier on these commodities and upload a photo of each to a secure platform. This photo is then fed to an AI algorithm that flags any images that have been submitted for more one than client, thus creating an opportunity for study implementers to investigate further (**Table 3,** **Example F**). Additional research could assess the effectiveness and value-add of using AI computer vision technology to assist with commodity tracking and accountability.

References

1. ClinicalTrials.gov. Pharmacy-based PrEP Delivery in Kenya. 2023. https://classic.clinicaltrials.gov/ct2/show/NCT05842122.

2. Kiptinness C, Naik P, Thuo N, Malen RC, Dettinger JC, Pintye J, et al. Online HIV prophylaxis delivery: Protocol for the ePrEP Kenya pilot study. Frontiers in Public Health. 2023;11:453. doi: 10.3389/fpubh.2023.1054559.

3. Sibanyaoni M CM, Ncube V, Pienaar J. POP INN: A key population led safe space for men-who-have-sex-with men (MSM) and the trans-community. HIV Nursing Mattesr. 2022;15.

4. The Aurum Institute. The ePrEP Programme. 2023. https://www.orderprep.co.za/copy-of-the-eprep-programme [Accessed January 5, 2024]

5. Clinicaltrials.gov. Pharmacy-based PrEP Delivery in Kenya (NCT05842122): National Institutes of Health; 2023. https://classic.clinicaltrials.gov/ct2/show/NCT05842122.

6. National AIDS & STI Control Program, Kenya Ministry of Health. Kenya HIV Testing Services Operational Manual 2022 Edition. 2022. https://www.prepwatch.org/wp-content/uploads/2023/04/Kenya-HTS-Manual.pdf

7. Sande LA, Matsimela K, Mwenge L, Mangenah C, Choko AT, d'Elbée M, et al. Costs of integrating HIV self-testing in public health facilities in Malawi, South Africa, Zambia and Zimbabwe. BMJ global health. 2021;6(Suppl 4):e005191. doi: 10.1136/bmjgh-2021-005191.

8. Shrivastava R, Fonjungo PN, Kebede Y, Bhimaraj R, Zavahir S, Mwangi C, et al. Role of public-private partnerships in achieving UNAIDS HIV treatment targets. BMC Health Serv Res. 2019;19(1):46. doi: 10.1186/s12913-018-3744-z.

9. National AIDS & STI Control Programme (NASCOP). Framework for the Implementation of Pre-exposure Prophylaxis of HIV in Kenya, Nairobi: National AIDS & STI Control Programme (NASCOP). (2017). https://www.prepwatch.org/wp-content/uploads/2018/12/Kenya_PrEP_Implementation_Framework.pdf[Accessed October 10, 2023].
